# Expanding the use of circulating microbiome in fish: contrast between the gut and blood microbiome of *Sebastes fasciatus*

**DOI:** 10.1093/ismeco/ycaf116

**Published:** 2025-07-11

**Authors:** Fanny Fronton, Arthur Gandin, David Deslauriers, Daniel Small, Dominique Robert, Yves St-Pierre

**Affiliations:** INRS—Centre Armand-Frappier Santé Technologie, 531 Boul. des Prairies, Laval, QC H7V 1B7, Canada; Institut des sciences de la mer, Université du Québec à Rimouski 310, allée des Ursulines, C.P. 3300 Rimouski, QC G5L 3A1, Canada; Institut des sciences de la mer, Université du Québec à Rimouski 310, allée des Ursulines, C.P. 3300 Rimouski, QC G5L 3A1, Canada; Fisheries and Oceans Canada, Institut Maurice-Lamontagne, 350 Route de la Mer, Mont-Joli, QC G5H 3Z4, Canada; Institut des sciences de la mer, Université du Québec à Rimouski 310, allée des Ursulines, C.P. 3300 Rimouski, QC G5L 3A1, Canada; INRS—Centre Armand-Frappier Santé Technologie, 531 Boul. des Prairies, Laval, QC H7V 1B7, Canada

**Keywords:** blood, gut, microbiome, 16S rRNA gene, fish, Sebastes, diet, Gulf of St. Lawrence

## Abstract

The study of microbiomes in fish populations offers vital insights for ecological and fisheries management, particularly in responses to environmental changes. Although traditional studies have concentrated on the gut microbiome, the emerging concept of a circulating blood microbiome suggests it may act as an early indicator of dysbiosis and various health conditions by reflecting transient bacterial DNA presence. In this study, we examined the gut and blood microbiomes of *Sebastes fasciatus* (Storer, 1854), a species of redfish of significant economic and ecological importance in the Gulf of St. Lawrence, to obtain critical information for health monitoring, pathogen detection, and ecological management in fisheries. Our results revealed that the gut and blood microbiomes of *S. fasciatus* have distinct bacterial DNA signatures, with significant differences in microbial diversity. Notably, although both microbiomes exhibited similar dominant genera, specific amplicon sequence variants varied significantly. Through a controlled experimental design, we found that the dietary impacts on microbiome composition were statistically significant yet minimal, suggesting that environmental factors play a more substantial role in shaping microbial communities. Finally, we report the presence of potential pathogens and opportunistic bacteria found exclusively in the blood microbiome. Our results highlight the blood microbiome's value as a sensitive health and environmental stress indicator, essential for sustainable fish population management. Integrating microbiome indicators can improve fisheries management and ecosystem sustainability, offering a model applicable to various marine species and environments.

## Graphical Abstract

**Graphical Abstract:**
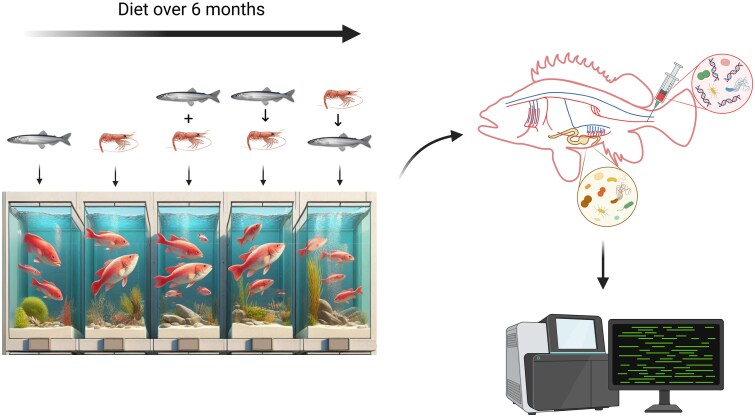

## Introduction

Approximately 38 trillion bacteria inhabit the human body [1], representing a diverse community that includes primarily transient or resident mutualistic or commensal species. This microbial community shifts rapidly due to abiotic and biotic factors, including diet, host genetics, and environmental factors [2–5]. Pioneering research studies have highlighted the significance of measuring the microbiome, defined here as the genetic fingerprint of the host's bacterial communities, for personalizing medical care, improving treatment outcomes, and informing clinical decisions across various health conditions. This includes studies showing that a diverse and balanced microbiome is essential for maintaining health. At the same time, an imbalance or alteration in the microbiome's composition (i.e. dysbiosis) can lead to a wide range of health issues, from gastrointestinal disorders to metabolic and mental health conditions [6–8].

Because the gut microbiota constitutes the body’s most densely populated bacterial niche [9], the gut microbiome has historically been the primary target for microbiome research. Many studies have focused on its dynamics and factors that influence its composition, notably diet, which can lead to changes in microbiome composition in 24–48 h [10–14]. In recent years, however, another microbiome niche has emerged as a promising health biomarker, providing a compelling complement to the gut microbiome. The concept of a circulating (blood) microbiome is relatively new yet controversial, given that blood has long been considered a sterile compartment, at least in healthy humans [15]. Recent studies suggest that the circulating microbiome reflects a temporary and sporadic passage of bacteria (or genetic material derived from bacteria) between compartments [16]. Through a process known as atopobiosis, bacteria and nucleic acids can enter the bloodstream from one niche and migrate to another [17, 18]. As blood circulates throughout the body, it is thought to collect and integrate signals from various microbiomes, developing a distinctive signature that may be used to detect early microbial shifts, dysbiotic conditions, and even pathogens [19–24]. Nowadays, the analysis of bacterial DNA in the blood is emerging as a valuable health biomarker across various pathologies, offering sensitivity and early detection comparable to that of the gut microbiome, with the added advantage of being less invasive for patients [20].

Although the potential of microbiome analysis for diagnostic and monitoring purposes has been developed mainly in humans, it is rapidly gaining interest in other species, including marine species [25–39]. In fish, monitoring the health status of populations is essential to ensure the balance of ecosystems, the sustainability of fisheries resources, food security and public health. It provides valuable information for the management of natural resources and the protection of aquatic habitats. Following a significant decline that led to the closure of fisheries in 1995, the *Sebastes fasciatus* and *Sebastes mentella*, once a prominent redfish species complex in the commercial fisheries of Eastern Canada, experienced a rapid population recovery due to strong recruitment events in 2011–2013. By 2019, the biomass of *S. mentella* was estimated to have reached a record high of 4.3 Mt, but it decreased to 2.3 Mt in 2023, likely due to density-dependent considerations. *S. fasciatus* biomass followed the same trend, although it is approximately one order of magnitude lower. These rapid and remarkable changes in the *Sebastes* population biomass highlight the importance of understanding the biological and ecological factors contributing to their health status for effective management and conservation strategies, most notably in response to climate change. In the present work, we first characterized and compared the gut and blood microbiome signatures of redfish using an experimental setting with controlled conditions. We further conducted an in-depth analysis of the effect of diet on the intestinal and blood microbiomes in *S. fasciatus* subjected to different diets. Finally, we have established that analyzing the circulating microbiome, a logistically simple method, is particularly effective as a monitoring tool for detecting potential pathogens.

## Material and methods

### Experimental design

For the experiment, we used *S. fasciatus* collected from the St. Lawrence estuary between 2019 and 2021 at Les Escoumins, Québec, Canada (48.317801°, −69.413287°) and maintained at the Fisheries and Ocean Canada’s Maurice Lamontagne Institute in Mont-Joli, Québec, Canada [40]. A total of 225 individuals, all aged between 7 and 8 years as determined by otolith measurements, were selected within the fork length range of 18 to 24 cm. They were randomly distributed into 15 circular 760 L tanks, with water pumped directly from the.

**Figure 1:**
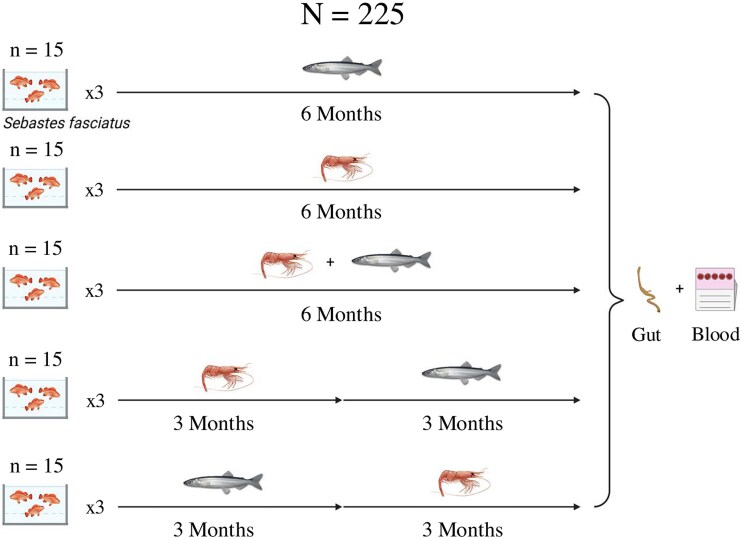
Experimental design and sampling strategy for the dietary study on *Sebastes fasciatus*. Figure illustrating the layout and protocol of a six-month dietary intervention study involving 225 *S. fasciatus*, distributed among 15 tanks at a density of 15 fish per tank. The study included five distinct dietary regimens, each replicated across three tanks. At the conclusion of the experiment, a subset of 90 fish (six from each tank) was selected for detailed analyses. From these selected fish, 88 gut samples were preserved by freezing at −20°C for subsequent analysis, and blood samples were collected from 89 fish and stored on FTA cards for DNA analysis. The figure details the tank distribution, dietary assignment, and sampling regimen used in the study.

St. Lawrence estuary. The salinity was adjusted by adding salt to the reservoir to maintain stability during the experiment. A custom-built thermal pump and unit from Gell’Air (Mont-Joli, Quebec) maintained the water temperature. The tanks were fully aerated. Temperature (5.0 ± 0.25°C) and salinity (28.6 ± 0.5 PSU) were measured daily and maintained throughout the entire experiment (6 months). Oxygen and pH were measured weekly with a handheld oxygen meter (FireSting GO2, PyroScience GmbH, Germany) and a pH multiparameter meter (H198194, Hanna Instruments, USA), with an average 101.5% ± 0.5% of air saturation (an average DO of 10.6 mg L^−1^) and pH of 7.7 ± 0.1. The fish were fed to satiation three times a week across five distinct dietary treatments over a 6-month period (Fig. 1). The fish were kept in a flow-through system, with 15 fish in each tank, and three replicates for each diet treatment (five treatments in total). These treatments were: (i) exclusively capelin (*Mallotus villosus, Cap*); (ii) exclusively Northern shrimp (*Pandalus borealis, shr*); (iii) a mixed diet, alternating daily between capelin and Northern shrimp (Mix); (iv) capelin for the first three months followed by Northern shrimp for the remaining three months (Cap-Shr); and (v) Northern shrimp for the first three months followed by capelin for the last 3 months (Shr-Cap). Frozen (−20°C) stocks of capelin and shrimp were obtained from local harvesters (Sainte-Flavie, QC, Canada) and scientific surveys led by Fisheries and Oceans Canada. Prey were cut into 2–3 cm pieces and thawed before being offered to redfish.

### Sampling

At the end of the experiment, following a 3-day starvation period, the redfish were anesthetized in a benzocaine solution tank before their individual length (fork length, mm) and wet body mass (g) were measured (Table 1). Blood was sampled with a sterile 3 ml syringe with a 22G × 1½ needle. The blood samples were collected from the caudal vein, above the anal fin and 1 cm under the lateral line. Blood was immediately stored on FTA cards and conserved in individual plastic bags with a desiccant. We failed to sample blood from one of the fish, reducing the number of samples to 89. For the gut microbiome, the fish were euthanized with an overdose of benzocaine solution followed by cervical dislocation. The entire digestive tract (stomach and intestine) was removed, placed in an individual plastic bag, and frozen at −20°C until further use. Two gut samples were damaged during the dissections and were withdrawn from the analysis, reducing the number of gut samples to 88. The wet mass of the gonads and liver were recorded for each fish as a proxy of maturity and health.

**Table 1:** Mean length and wet body mass of *S. fasciatus* individuals.

	n	Length (cm) mean ± SE	Weight (g) mean ± SE
Male	66	22.2 ± 0.2	179.6 ± 4.4
Female	21	22.9 ± 0.3	200.7 ± 9.6
Unknown	2	22.8 ± 1.0	185.5 ± 12.7
Total	89	22.4 ± 0.2	184.7 ± 4.1

### DNA extraction, amplification, and sequencing

All DNA extraction and purification procedures were conducted in a controlled clean room, where pressure, temperature, and humidity were maintained to minimize contamination. DNA from blood samples was isolated as previously described by Fronton et al. (2023, 2024). A negative control was conducted on FTA cards during sampling. The excess material inside the gut samples was squeezed out with sterile tweezers, and the last 2 cm of gut samples were used for DNA extraction. For the prey microbiome analysis, two entire capelin or two entire shrimp were cut into 2–3 cm pieces and pooled to mix in bleach and ethanol-cleaned mixer with 50 ml of RNAse and DNAse-free water. DNA from redfish gut and prey was extracted using the QIAamp DNA Investigator Kit (Qiagen, Toronto, ON, Canada) according to the manufacturer’s protocol. Amplification and sequencing were adapted, and a Semi-Nested PCR was performed to maximize the number of 16S rRNA gene reads for the blood microbiome. A comparison of microbiome diversity and ASV characterization was made between PCR, Semi-Nested PCR, and Nested PCR was made using previous results to ensure no biases were introduced (unpublished data). A Semi-Nested PCR was conducted by Génome Québec on the bacterial 16S rRNA gene for the blood microbiome. Genome Quebec performed a negative control using PCR water to ensure that no contamination was introduced. For the first PCR, the V1-V4 region was targeted with the 27F (5’AGAGTTTGATCMTGGCTCAG3’) and 805R (5’GACTACHVGGGTATCTAATCC3’) primers. The second PCR was performed on the V3-V4 region using the 341F (5’CCTACGGGNGGCWGCAG3’) and 805R primers. PCR amplification was performed directly on the V3-V4 regions with the same primers for the gut microbiome. Sequencing was conducted on an Illumina platform, NextSeq PE300 10 M reads. Two samples failed to be sequenced from the gut DNA extraction, reducing the number of redfish gut microbiomes analyzed to 86. The raw data files are publicly available on the NCBI Sequence Read Archive (PRJNA1212171).

### 16S rRNA data processing and analysis

Illumina sequence data (FASTQ files) were trimmed using *Cutadapt* (version 4.0) [41]. The 16S rRNA (V3-V4) amplicon sequence variants (ASVs) were generated with the DADA2 pipeline (version 1.16.0; [42]) and subsequently within the R environment (R version 4.1.1, Team (2021)). Forward and reverse reads were then trimmed, filtered, and truncated with the filterAndTrim function. The error model (maxEE) was calculated for forward and reverse reads, and low-quality reads were removed. After denoising and merging, chimeric sequences (bimeras) were removed from the datasets. The minimum and maximum lengths were set at 400 bp and 428 bp, respectively. All reads had an average quality score of ≥30. The Version 19 of the RDP 16 classifier was used for the ASV assignment. Software packages *phyloseq* (1.48.0), *microbiomeSeq* (0.1), *microbiomeMarker* (1.10.0), and *vegan* (2.6.8) were used to characterize the microbial communities [43–46]. The graphics were created using the ggplot2 package *(*version 3.5.1).

Bacterial taxonomic α-diversity (intrasample) was estimated using the richness, Shannon, Simpson and Pielou indices with the R package *microbiome* (version 1.14.0). Variations in bacterial α-diversity and taxa abundances between the two blood and gut microbiomes were assessed using the Wilcoxon-Mann–Whitney test since there was no normal distribution of values. The *P*-value (*P*) was considered significant for the Wilcoxon-Mann–Whitney test at *P* < .05.

The β-diversity (inter-sample) was estimated using phylogenetic weighted UniFRac dissimilarities assessed by principal coordinates analysis (PCoA). Differences in community composition were tested using permutational multivariate analysis of variance (PERMANOVA) for weighted UniFRac indices with 10 000 permutations, as implemented in the R *vegan* package or the *pairwise Adonis* package (0.4).

The redundancy analysis (RDA) was performed on the standardized (rclr transformation) number of reads for each ASV in each matrix and the environmental and physiological variable matrix [47]. The collinearity between variables was validated with a Pearson correlation, and weight and length were the only variables correlated. An Analysis of Compositions of Microbiomes with Bias Correction 2 (ANCOM-BC2) was done between the diet treatment and basin signatures using the *ANCOMBC* packages (version 2.6.1) [48, 49]. A classical ANCOM-BC and a Differential abundance analysis with the *DESeq2* package (version 1.44.0) [50] were done between the niche signatures. For all the ANCOM-BC and DESeq analyses, only the significant ASV or genera were kept. Differences were considered statistically significant at *P* < .05.

A ratio was calculated for differential abundant ASV in each niche with:

When $\overline{x}{\left({ASV}_i\right)}_{gut}>\overline{x}{\left(A{SV}_i\right)}_{blood}:$


$$ \frac{\overline{x}{\left({ASV}_i\right)}_{gut}}{\overline{x}{\left(A{SV}_i\right)}_{blood}+1} $$

When $\overline{x}{\left({ASV}_i\right)}_{gut}<\overline{x}{\left(A{SV}_i\right)}_{blood}:$


$$ \frac{\overline{x}{\left({ASV}_i\right)}_{blood}}{\overline{x}{\left(A{SV}_i\right)}_{gut}+1}\times \left(-1\right) $$

Data analyses were performed in R (version 4.4.1) with RStudio (version 2024.12.0).

### Semi-nested PCR for the detection of *Ralstonia picketti*

To confirm the presence of *Ralstonia picketti*, we employed a semi-nested PCR targeting the 16S rRNA gene. In the first amplification cycle, we used primers 27F (V1 region; AGAGTTTGATCMTGGCTCAG) and 805R (V4 region; GACTACHVGGGTATCTAATCC) on DNA extracted from blood and gut samples. The second amplification cycle utilized the specific primer RpF (ATGATCTAGCTTGCTAGATTGAT) for *Ralstonia picketti* and the 805R primer [51, 52]. The first cycle consisted of an initial denaturation at 95°C for 5 minutes, followed by 20 cycles of denaturation at 94°C for 1 minute, annealing at 60°C for 1 minute, and elongation at 72°C for 1 minute and 30 seconds, concluding with a final extension at 72°C for 15 minutes. The second cycle included denaturation at 95°C for 5 minutes, followed by 20 cycles of denaturation at 94°C for 30 seconds, annealing at 58°C for 30 seconds, and elongation at 72°C for 1 minute, with a final extension at 72°C for 10 minutes. PCR products were analyzed via electrophoresis on 1.5% agarose gels containing SYBR™ Safe DNA Gel Stain. Amplicons were sequenced using the Sanger method by Genome Québec (Montreal, Québec, Canada).

## Results

### Gut and blood microbiome characterization

The gut and blood microbiome signatures were obtained by sequencing the V3-V4 hypervariable regions of the 16S rRNA gene. Approximately 2.5 million raw reads were obtained after quality filtering in the gut microbiome's case. Sequence counts per sample were 10 591 to 62 650 reads, with the mean read counts per individual being 27 727.22 ± 1131.98 reads. As for the observed richness, 4179 different ASVs were detected in the gut microbiome (mean = 75.49 ± 5.29 per individual). Approximately 1.5 million raw reads were obtained for the blood microbiome, ranging from 3856 to 32 038 reads per sample, with an average of 17 212.75 ± 471.25. In both cases, the ASV accumulation curves indicated that sequencing depth was sufficient to capture the full ASV diversity of both microbiome niches (Fig. S1).

**Figure 2:**
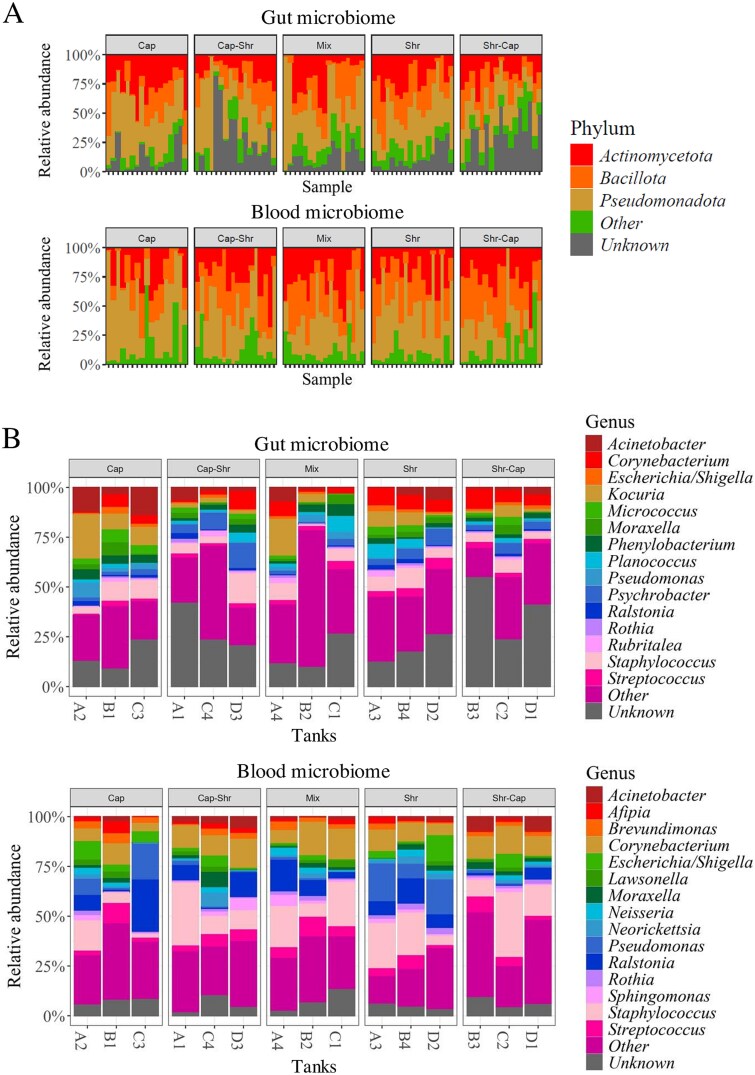
Relative abundance of the main taxa in the blood and gut microbiome. (A) Individual relative abundance of the main phylum of the Redfish’s microbiome, prevalence = 50%, detection threshold = 1%, n (gut) = 86 and n (blood) = 89. (B) Mean by tank of the relative abundance of the main genera of the Redfish’s microbiome, prevalence = 30%, detection threshold = 1%, n (gut) = 86 and n (blood) = 89.

Regarding the bacteria found in the gut and blood microbiomes, we found no significant differences at the phylum level between both niches, although the percentage of DNA of unknown origin was higher in the gut microbiome (Fig. 2A). However, we found significant differences in the microbiome signatures at the genus level. In the gut, the microbiome was dominated by DNA from *Staphylococcus* (mean = 7.03% ± 0.98%), *Kocuria* (mean = 6.50% ± 1.78%), which was not found in the blood microbiome, and *Corynebacterium* (mean = 5.65% ± 0.86%) (Fig. 2B). This dominance by *Staphylococcus* (mean = 15.65% ± 2.62%) and *Corynebacterium* (mean = 10.92% ± 1.03%) was also observed in the blood microbiome. However, the genus *Ralstonia* was more prevalent in the blood microbiome, with a relative abundance of 8.09% ± 1.91% compared to 1.10% ± 0.26%. Other genera specific to the circulating microbiome included *Afipia*, *Brevundimonas, Lawsonella, Neisseria, Neorickettsia*, and *Sphingomonas* (Fig. S2).

### Differences between the gut and blood microbiomes

**Figure 3:**
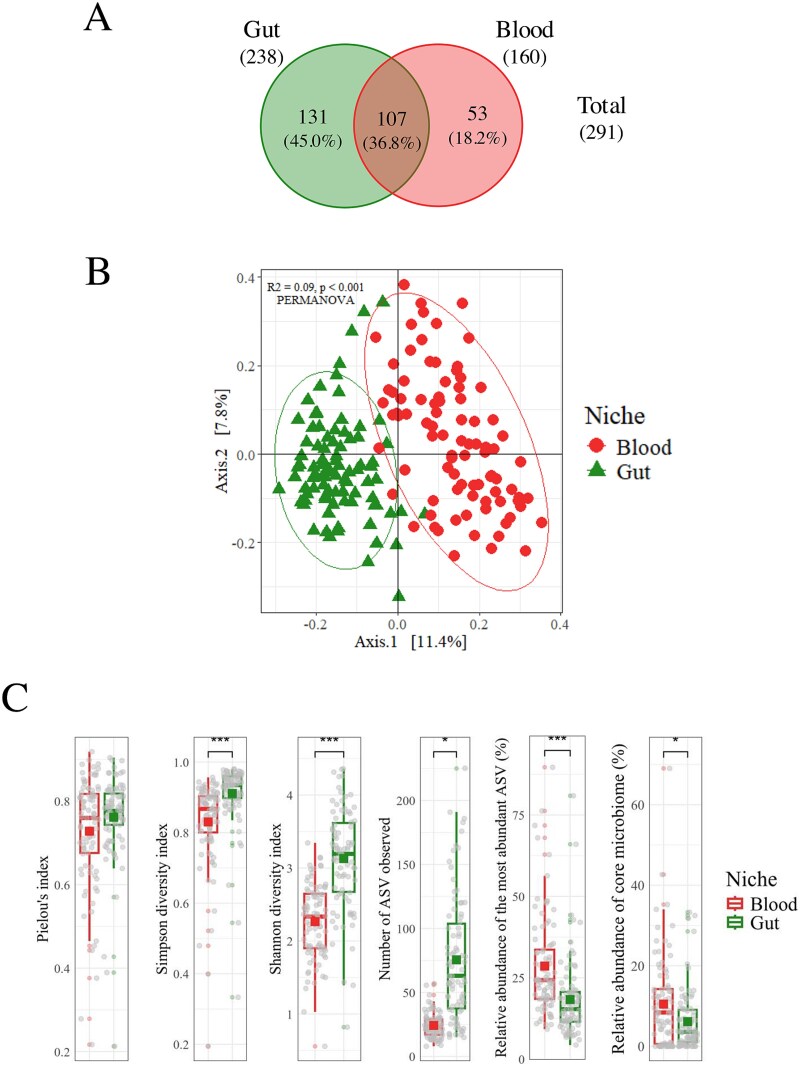
Differences between the blood and gut microbiomes. (A) Unique and shared genera. (B) PCoA plot of the microbial communities found in two distinct biological niches, ellipses represent an estimation of a t-distribution around the centroids, n (gut) = 86 and n (blood) = 89. (C) A comparison of diversity indices and relative abundance metrics of microbial communities between the two biological niches. Prevalence = 50%; detection threshold = 1%; n (gut) = 86 and n (blood) = 89. 9; ^*^P < .05, ^**^P < .01, ^***^P < .001.

Overall, we identified 238 genera in the gut, of which 141 (55.0%) were exclusive to this niche, including *Micrococcus, Phenylobacterium, Planococcus, Psychrobacter*, and *Rubritalea* (Fig. 3A). In contrast, 160 genera were identified in the blood, with 53 (33.1%) being unique to blood samples. Overall, 107 genera (36.8%) were also shared between the two environments, indicating some commonalities in microbial communities and significant niche-specific diversity. A PCoA of microbiome compositions (using Weighted UniFrac distances) confirmed two distinct clustering patterns between gut and blood samples, with a statistically significant separation between the two niches (PERMANOVA: R^2^ = 0.09, *P* < .001) (Fig. 3B). Further analysis revealed significant differences in microbial diversity between the gut and circulating microbiomes (Fig. 3C). Specifically, the number of ASVs, the Shannon diversity index, and the Simpson diversity index were all markedly higher in the gut microbiome. Yet, both microbiomes displayed similar evenness levels, as indicated by Pielou’s evenness index. We also found that the core microbiome analysis indicated low dominance among the ASVs. The most abundant ASV represented 18.29% ± 1.32% of the total ASVs in the gut microbiome, compared to 28.58% ± 1.64% in the circulating microbiome. These percentages reflect the relative abundance of the most dominant ASV within each microbiome, indicating a significant difference in microbial dominance between the two environments (*P* < .001). Additionally, the core microbiome's relative abundance was 6.13% ± 0.80% in the gut and 10.48% ± 1.22% in the circulating microbiome (*P* < .05). These findings suggest that while the gut microbiome is more diverse, both environments maintain a balanced distribution of microbial taxa with low dominance.

**Figure 4:**
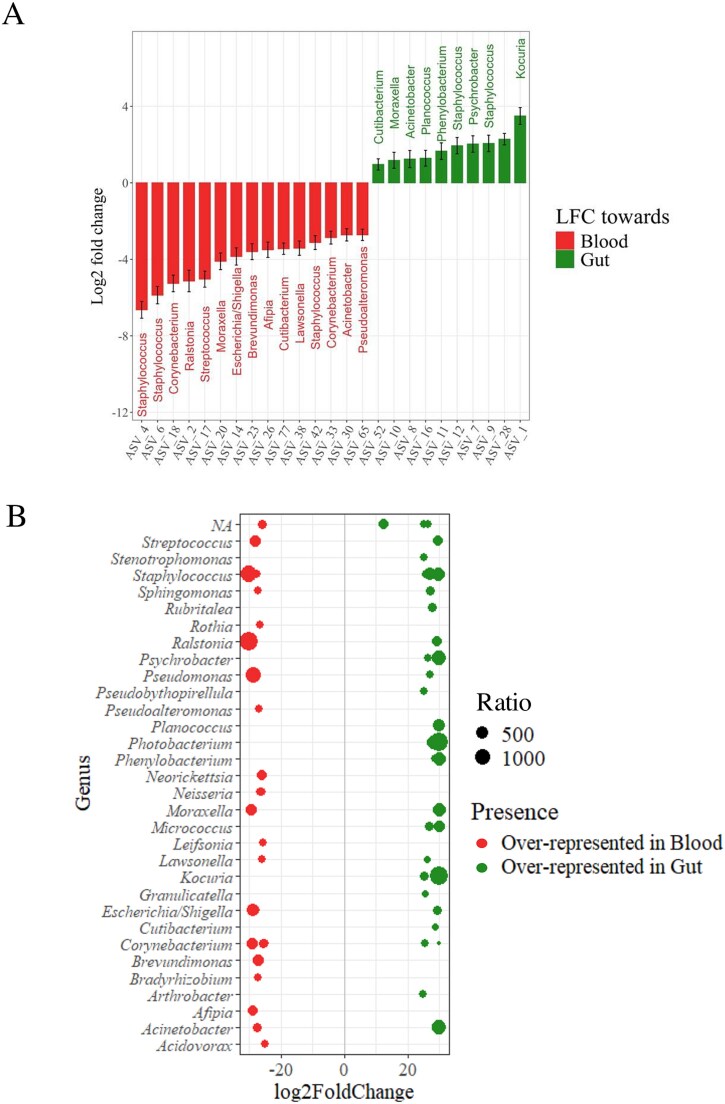
Significant differences in ASVs observed between the niches. (A) Differential abundance of ASVs between blood and gut niches as indicated by ANCOM-BC LogFoldChange values. (B) Bubbleplot of the ASV with a significant differential abundance between niches. Each point represents an ASV. The larger a point appears on the graph, the greater the disparity in ASV abundance between the niches. Gut (n = 86); Blood (n = 89).

An ANCOM-BC analysis was conducted to further explore the differences between the gut and blood microbiomes at the ASV level (Fig. 4A, Table 2). We observed that while *Staphylococcus* ASVs were identified in both microbiomes, they represented distinct ASVs, suggesting that different species or strains of *Staphylococcus* may dominate each niche. Additionally, *Corynebacterium*, present in both microbiomes, showed significant enrichment in the circulating microbiome, particularly ASV18 and ASV33, which together accounted for approximately one-third of the total *Corynebacterium* abundance in the blood (mean relative abundance: ASV18 = 2.38% ± 0.42%, ASV33 = 1.17% ± 0.41%). Furthermore, *Kocuria* and *Ralstonia*, previously recognized as specific to the gut and blood microbiomes, respectively, exhibited significant differences in abundance in their respective niches. The DESeq2 analysis corroborated the findings from the ANCOM-BC (Fig. 4B), revealing pronounced ASV-level differences: *Ralstonia* had a mean relative abundance ratio of −1424.81 in the circulating microbiome, while *Kocuria* displayed a ratio of 1492.47 in the gut. The *Ralstonia* ASV was identified in the pipeline as *Ralstonia pickettii* (ASV2), a potential human pathogen. Additional potential human pathogens were detected solely in fish blood through the ANCOM-BC and DESeq analyses, including *Corynebacterium tuberculostearicum*, *Moraxella osloensis*, and *Acinetobacter junii*. These results suggest that analyzing the blood microbiome can be a valuable tool for detecting pathogens, as it may reveal the presence of opportunistic bacteria.

**Table 2:** ANCOM-BC Results of the comparison of gut and circulating microbiome.

ASV #	LFC	SE	W	p-value	q-value	Genus	Species
ASV_1	3.50	0.44	7.94	1.99E-15	4.60E-15	*Kocuria*	
ASV_28	2.28	0.30	7.63	2.42E-14	5.28E-14		
ASV_9	2.06	0.43	4.78	1.74E-06	3.57E-06	*Staphylococcus*	
ASV_7	2.03	0.43	4.73	2.29E-06	4.46E-06	*Psychrobacter*	*pulmonis*
ASV_12	1.94	0.43	4.54	5.54E-06	1.02E-05	*Staphylococcus*	
ASV_11	1.66	0.43	3.84	1.24E-04	2.19E-04	*Phenylobacterium*	
ASV_16	1.29	0.41	3.12	1.83E-03	2.95E-03	*Planococcus*	
ASV_8	1.24	0.45	2.76	5.78E-03	8.55E-03	*Acinetobacter*	*lwoffii*
ASV_10	1.18	0.42	2.81	5.02E-03	7.73E-03	*Moraxella*	*osloensis*
ASV_52	0.96	0.29	3.27	1.08E-03	1.81E-03	*Cutibacterium*	*acnes*
ASV_65	−2.72	0.30	−9.18	4.12E-20	1.90E-19	*Pseudoalteromonas*	
ASV_30	−2.72	0.32	−8.53	1.41E-17	3.48E-17	*Acinetobacter*	*junii*
ASV_33	−2.87	0.34	−8.55	1.28E-17	3.39E-17	*Corynebacterium*	
ASV_42	−3.12	0.36	−8.57	1.03E-17	3.19E-17	*Staphylococcus*	
ASV_38	−3.42	0.37	−9.19	3.92E-20	1.90E-19	*Lawsonella*	
ASV_77	−3.45	0.30	−11.60	4.16E-31	3.08E-30	*Cutibacterium*	*acnes*
ASV_26	−3.50	0.40	−8.69	3.63E-18	1.34E-17	*Afipia*	*birgiae*
ASV_23	−3.60	0.42	−8.66	4.90E-18	1.65E-17	*Brevundimonas*	
ASV_14	−3.85	0.45	−8.56	1.12E-17	3.19E-17	*Escherichia/Shigella*	
ASV_20	−4.11	0.43	−9.45	3.35E-21	2.07E-20	*Moraxella*	*osloensis*
ASV_17	−5.04	0.42	−11.98	4.26E-33	3.94E-32	*Streptococcus*	
ASV_2	−5.14	0.56	−9.15	5.80E-20	2.38E-19	*Ralstonia*	*pickettii*
ASV_18	−5.26	0.43	−12.13	7.60E-34	9.37E-33	*Corynebacterium*	*tuberculostearicum*
ASV_6	−5.88	0.45	−12.97	1.91E-38	3.54E-37	*Staphylococcus*	
ASV_4	−6.64	0.44	−15.12	1.12E-51	4.13E-50	*Staphylococcus*	

### Effect of the diet on the host’s microbiome

**Figure 5:**
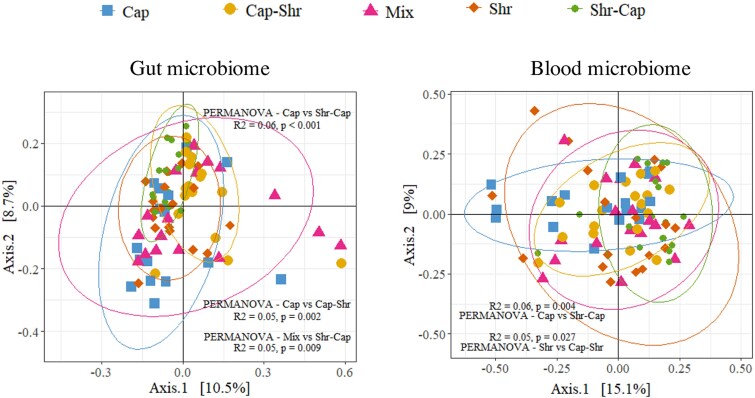
PCoA of microbiome diversity in different treatment groups. The *left panel* shows a PCoA plot that displays the microbial diversity in the gut across various treatment groups, visualized along two principal coordinate axes explaining 10.5% and 8.7% of the variance, respectively. The *right panel* illustrates the microbial diversity in the circulating microbiome, with axes explaining 15.1% and 9.0% of the variance, respectively. Ellipses represent an estimation of a t-distribution around the centroids of each group. Statistical significance between groups is indicated, with PERMANOVA results showing variations in microbial composition between treatments.

To examine whether the 6-month diet impacted the microbiomes, we first investigated differences in microbiome composition among dietary treatments using a PCoA (Fig. 5). Our PERMANOVA analysis revealed a significant effect of diet on gut microbiome composition. However, the R^2^ values obtained were low (< 0.06), indicating that the influence of diet on gut and blood microbiomes, while statistically significant, appeared minimal. The lack of clear clusters in the PCoA supports this conclusion. Furthermore, the replicates for each diet treatment yielded divergent results, including statistically significant differences (Fig. S3 and S4). This complicates the interpretation of the data, as the grouped samples with the same diet were not uniform compared to those of another diet. Moreover, no ASV was identified in the ANCOM-BC2 analysis that differed between the diet treatments.

To provide additional insights into our analysis, we conducted an RDA. Our results indicated that the niche, diet treatment, and basin factors were statistically significant (Table 3), collectively explaining ~5% of the variance (adjusted R^2^ = 0.0527). Separate RDA analyses for each niche revealed that basin and diet treatment significantly influenced microbial composition in both the gut and circulating microbiomes, with a notably greater impact from the basin factor, as reflected by higher variance and degrees of freedom (Table S1). In both cases, however, the models accounted for a modest level of variance (blood: adjusted R^2^ = 0.042; gut: adjusted R^2^ = 0.046), suggesting these factors represent only a limited portion of the variability in microbiome profiles. Furthermore, in some cases, the gut and blood microbial composition significantly differed between two basins of the same diet treatment (Fig. S5 and S6), complicating the interpretation. In conclusion, while the 6-month diet demonstrated a statistically significant impact on microbiome composition in certain instances, the low R^2^ values, lack of results in the ANCOMBC2, and the modest variance explained by dietary and environmental factors, along with a basin effect, indicate that the actual influence of diet on gut and blood microbiomes is minimal and complex. The variation in compositions between tanks of the same diet seems to impact the significance of the results across diets.

**Table 3:** RDA results of the measured factors analyzed with gut and circulating microbiome. Explained variance is described in percentage in brackets

Factors	Degree of freedom	Variance	F statistics	p-value
Length at the start	1	0.304 (0.51%)	0.9322	0.6513
Length at the end	1	0.339 (0.57%)	1.0375	0.3996
Weight at the start	1	0.277 (0.47%)	0.8482	0.8352
Weight at the end	1	0.358 (0.60%)	1.0954	0.2877
Gonad’s weight	1	0.353 (0.59%)	1.0824	0.2597
Liver’s weight	1	0.335 (0.56%)	1.0250	0.4066
Length growth per day	1	0.928 (1.56%)	0.9742	0.5094
IGS	1	0.280 (0.47%)	0.8576	0.8172
IHS	1	0.310 (0.52%)	0.9490	0.5584
Fulton’s K	1	0.301 (0.51%)	0.9224	0.6783
Sex	2	0.606 (1.02%)	0.9284	0.5405
Diet treatment	4	1.780 (2.99%)	1.3632	0.0019 (**)
Basin	10	3.879 (6.52%)	1.1879	0.0009 (***)
Niche	1	2.455 (4.12%)	7.5178	0.0009 (***)
Residuals	144	47.019 (78.99%)		

## Discussion

Our study aims to compare the gut and blood microbiomes of *S. fasciatus* to understand how these two distinct microbiomes may be influenced by dietary and environmental factors. Studying the gut and blood microbiomes of *S. fasciatus* and their variation in response to diet and environmental factors is crucial for understanding how climate change could impact the health and resilience of this ecologically and economically important fish species, thereby informing sustainable fisheries management and conservation strategies. Our results revealed that although both microbiomes were dominated by similar bacterial genera, specific ASVs differed significantly, emphasizing the unique microbial composition of each niche. Both microbiomes were also to be relatively stable to dietary changes over several months, even with a diet consisting of shrimp and capelin, two marine species with distinct nutritional properties. Finally, we also demonstrated that analyzing the blood microbiome, which reflects the release of bacterial DNA from various host tissues, is an interesting tool for detecting potential pathogens. The detection of potential pathogens was a secondary outcome resulting from the broader microbial profiling. This study highlights the importance of circulating microbiome analysis as an innovative method for detecting potential pathogens, while also enhancing our understanding of microbial interactions and their stability in response to dietary variations. This approach could have significant implications for fish health and marine resource management.

As blood circulates, it can encounter DNA fragments released from various sources, such as minor skin lesions or oral activities like tooth brushing in humans. This phenomenon may explain why prey microbiomes cluster closely with the circulating microbiome as it gathers bacterial DNA from all niches within the body. This has been well documented regarding circulating cell-free DNA (ccfDNA) fragments released by apoptotic cancer cells [53–55]. Today, the analysis of ccfDNA enables the detection of primary tumors, which can be challenging to identify clinically, and provides information that allows clinicians to adapt and monitor specific treatments. Studies on ccfDNA have demonstrated that blood contains DNA fragments from diverse sources, including microorganisms. These observations have led to the concept of the circulating microbiome.

Although the concept of a blood microbiome is met with skepticism within the scientific community—stemming primarily from the longstanding belief that healthy host blood is sterile—our study navigates this contention [15, 20, 56]. Our study does not focus on the presence of live bacteria, or microbiota, which is commonly referred to as the assemblage of live microorganisms present in a defined environment, including bacteria, archaea, fungi, and viruses. It rather focused on the microbiome, which refers to the presence of bacterial DNA. This is why we have performed the standard 16S rRNA gene sequencing. This is why our methodology utilized just a single drop of blood, allowing it to target DNA fragments of bacteria, circumventing traditional concerns regarding the viability of bacteria in the bloodstream. Notwithstanding the controversy surrounding the presence of healthy bacteria in the blood, it is thus essential to clarify that the concept of a circulating microbiome, at least in this case, is based not on the analysis of bacteria in the blood but instead on DNA fragments of bacterial origin, as in the case of ccfDNA. The prevailing view is that these fragments may result from transient and sporadic translocation from several tissues, including infected tissues and bacteria that constitute the normal microbial flora, such as the intestine, oral mucous membranes, and other organs, including the brain [16, 33]. Therefore, it is not surprising that the signature of the blood microbiome differs from that of the gut. These observations support the “Transient Bacterial DNA Hypothesis for the Circulating Microbiome” as a guiding paradigm, wherein the circulating microbiome comprises DNA fragments rather than whole bacteria, reflecting a digital snapshot of bacterial DNA originating from various body sites. These DNA fragments can enter the bloodstream through cellular turnover, apoptosis, or minor breaches in the barriers separating commensal or pathogenic bacteria from the bloodstream. Detecting specific bacterial DNA in the blood may reflect pathogenic events and normal biological processes, including the migration of DNA from tissues where bacteria are generally present.

In our methodology, blood samples are collected via puncture, similar to standard clinical procedures. Concerns may arise regarding potential contamination with bacterial DNA from skin and muscle tissues during the sampling process. However, several factors mitigate this risk in our study. First, the bacterial biomass in muscle tissue is significantly lower than that in blood, reducing the likelihood that DNA from muscle bacteria substantially influences our results. Furthermore, any bacterial DNA fragments present on the skin or in the muscle are likely to be minimal and would represent a consistent background level across all samples, thus not disproportionately affecting the analysis. It is also important to note that the procedure used for collecting blood samples from fish closely mirrors that used in human clinical settings, where the risk of contamination is well understood and controlled. Therefore, we assert that the integrity of our data is not compromised by the sampling method, and the bacterial DNA we analyze predominantly reflects the microbiome associated with the blood and immune cells rather than external contamination.

Our study shows that dietary treatment had a minimal impact on the blood and gut microbiomes of redfish. Although significant differences appear in the PCoA analysis, the points are mixed, indicating homogeneity of the microbiomes. Furthermore, the replicates (basins) for each diet present divergent results, some of which are significantly different. This complicates data interpretation because the pools fed with the same diet are not uniform compared to those of another diet. While existing literature suggests that short-term and long-term dietary regimes alter the composition and functionality of the gut microbiota, which is a primary factor influencing gut microbial composition [14, 57, 58], the observed differences between shrimp and capelin diets over six months were insufficient to drive distinct microbial signatures. The nutritional profiles of capelin and shrimp as fish feed are very different. Capelin offers higher omega-3 fatty acids than shrimp, while shrimp provide a different amino acid profile and generally have higher levels of carbohydrates [59–61]. Capelin is also considered more digestible due to its high protein and low-fat composition, which can lead to better nutrient absorption in fish compared to shrimp, which contains chitin, a less digestible component [62, 63]. When we examined the microbiomes of each prey, we found that the differences in the microbiome composition between capelin and shrimp were evident. Some bacterial genera appear to be more prevalent in one type over the other, indicating that the intrinsic properties of each organism (such as diet, habitat, or physiology) might influence the microbial communities they host (Fig. S7). For example, *Escherichia/Shigella* showed a higher relative abundance in some shrimp samples than in capelin, while genera like *Rubritalea* and *Rothia* showed a higher abundance in capelin. However, despite these differences, the microbiome of the prey had little effect on the gut microbiome. This limited influence is likely due to the complex interplay of factors such as digestion, host immune response, and existing microbial interactions within the host, which can modulate the impact of diet on the microbiome. Studies in humans have shown that all these factors are known to impact the gut microbiome [64–66]. It is plausible that the nutritional compositions of these diets were not divergent enough to induce significant shifts within the experiment's timeframe. Rapid changes in the microbiome are generally observed in response to diets that differ markedly, such as animal and plant-based diets [10–14].

In our study, the minimal shift in the microbiome was similar to the differences between tanks, as shown by our RDA, which indicated that while diet is statistically significant, its effect is considerably smaller than that of the basin factor, even with all tanks sharing a common water supply. Each tank serves as a distinct microenvironment and can reflect subtle shifts in the microbial composition, reflecting each tank's specific interactions and conditions. This “co-housing effect” on the microbiome has been well documented in mice model systems, where individuals in close proximity exhibit similar microbiota due to microbial exchange through direct contact, airborne transmission, or shared handling by caretakers [67–69]. The tank effect we observed was particularly evident in the gut microbiomes from the mixed diet group, where distinct bacterial structures were observed within tanks. Yet, individuals showed remarkably similar profiles. The ability of the microbiome to remain stable in the face of dietary variations could offer a form of resilience to these populations, an essential factor to consider in adaptation strategies to climate change. While the changes were minimal, they underline the significance of considering environmental and social factors in shaping the microbiome of redfish in controlled settings. It also suggests that environmental factors should be emphasized over dietary changes to monitor the health level of redfish populations. The microbiome of the water in the tank, however, is likely to have a limited impact on the host microbiome, as previous findings have shown that the microbiomes of water and hosts are significantly different [70, 71].

*S. fasciatus* are naturally gregarious, forming large schools in the wild, a behavior likely intensified by their increased abundance in the Gulf of St. Lawrence. This schooling behaviour could facilitate greater bacterial sharing than less gregarious species, potentially heightening vulnerability to pathogen spread [70, 71]. Our results with *R. pickettii* and other potential pathogens exemplify this hypothesis. Our preliminary results, using PCR primers specific for *R. pickettii*, have confirmed the presence of DNA fragments from this bacteria in the blood (Fig. S8, Supplementary files S1 and S2). *R. pickettii* is an opportunistic pathogen linked to nosocomial outbreaks, typically introduced through contaminated water or saline. It has been isolated from soil, freshwater, and marine ecosystems [72–74]. While this bacterial species was reported in fish gastrointestinal and skin microbiomes, no disease symptoms have been associated with it [75–78], suggesting it may function as a commensal or mutualistic bacterium within aquatic ecosystems. We also detected DNA derived from several other human-associated potential pathogens, including *C. tuberculostearicum*, *M. osloensis*, and *A. junii*, which were detected in the blood microbiome [79–81]. Together, these findings illustrate the potential of the blood microbiome as a valuable monitoring tool for identifying potential pathogens in fish populations. In humans, the blood microbiome serves as a useful biomarker for detecting potential pathogens, accelerating diagnostics and improving the timing of medication for the patient [82–85]. However, while our findings suggest the potential utility of blood microbiome analysis in pathogen detection, actual efficacy in a clinical or health management context will require further investigation under controlled conditions where health status and infection are directly monitored. Future studies should aim to correlate microbiome changes with confirmed pathogenic infections and health declines to validate this approach. It is also important to approach the detection of bacterial DNA cautiously, as it does not inherently indicate active infection or a direct health risk to humans or other hosts, including fish. While *Ralstonia* is known to be pathogenic in humans, to our knowledge, its pathogenicity in fish has not been documented. Further targeted research is necessary not only to confirm its presence but also to fully elucidate the pathogenic potential of these bacteria and their implications for both fish health and human safety.

## Supplementary Material

Supplementary_Information_ycaf116

Suppl_files_1_Data_seb_ycaf116

Suppl_files_2_Fish_R_picketti_ycaf116

## Abbreviations

*S. fasciatus, Sebastes fasciatus*; *Sebastes mentella*, *S. mentella*; cap, capelin; shr, shrimp; PCoA, Principal coordinates analysis; RDA, Redundancy analysis; ASV, Amplicon sequence variant; PSU Practical salinity unit;, ANCOM-BC, Analysis of Compositions of Microbiomes with Bias Correction; PERMANOVA, Permutational multivariate analysis of variance.

## Data accessibility

Metagenome sequencing data have been deposited in the NCBI Sequence Read Archive under the Bioproject PRJNA121217.
