# Supplementary material for: Expanding the use of circulating microbiome in fish: contrast between the gut and blood microbiome of Sebastes fasciatus
Source: ISME Commun. 2025 Jul 11;5(1):ycaf116. doi: 10.1093/ismeco/ycaf116 (PMC12342392; doi:10.1093/ismeco/ycaf116)
Supplement: Supplementary_Information_ycaf116 [file supplementary_information_ycaf116.pdf]

Supplementary figures

Expanding the Use of Circulating Microbiome: Contrast between the Gut and Blood Microbiome of *Sebastes fasciatus*.

Fanny Fronton, Arthur Gandin, David Deslauriers, Daniel Small, Dominique Robert, Yves St-Pierre.

Figure S1

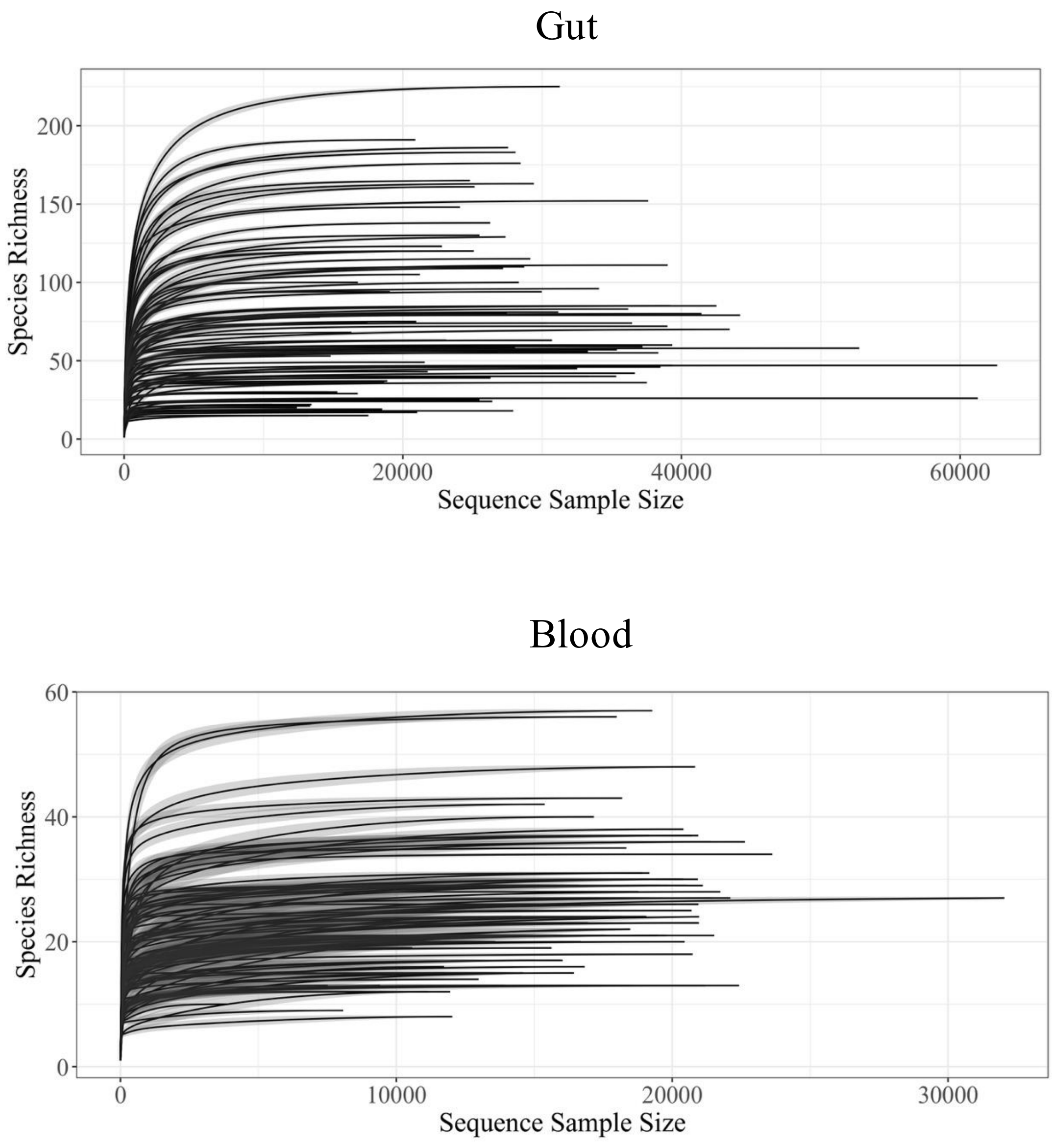

Figure S1: Sequencing depth curves based on the number of reads (sample size) and the number of ASVs (richness) for each sample. Each line represents one sample. Gut microbiome, n_{gut}= 86; Blood microbiome, n_{blood}=89.

Figure S2

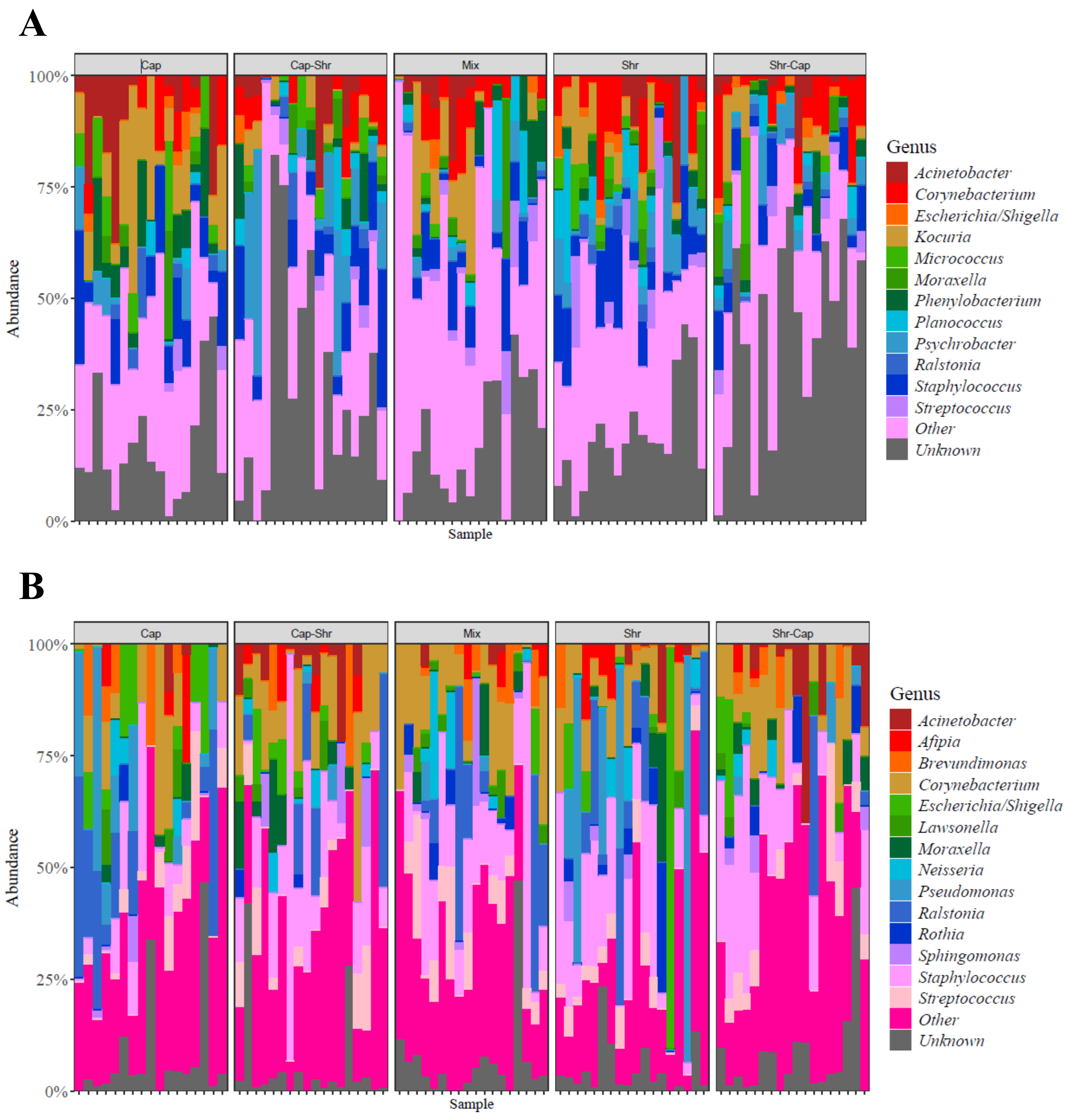

Figure S2: Individual relative abundance of the main shared genera in the Redfish's microbiomes for each diet treatment. (**A**) gut microbiome, n_{gut}=86, prevalence = 20%, detection threshold = 1%. (**B**) blood microbiome, n_{blood}=89, prevalence = 20%, detection threshold = 1%.

Figure S3

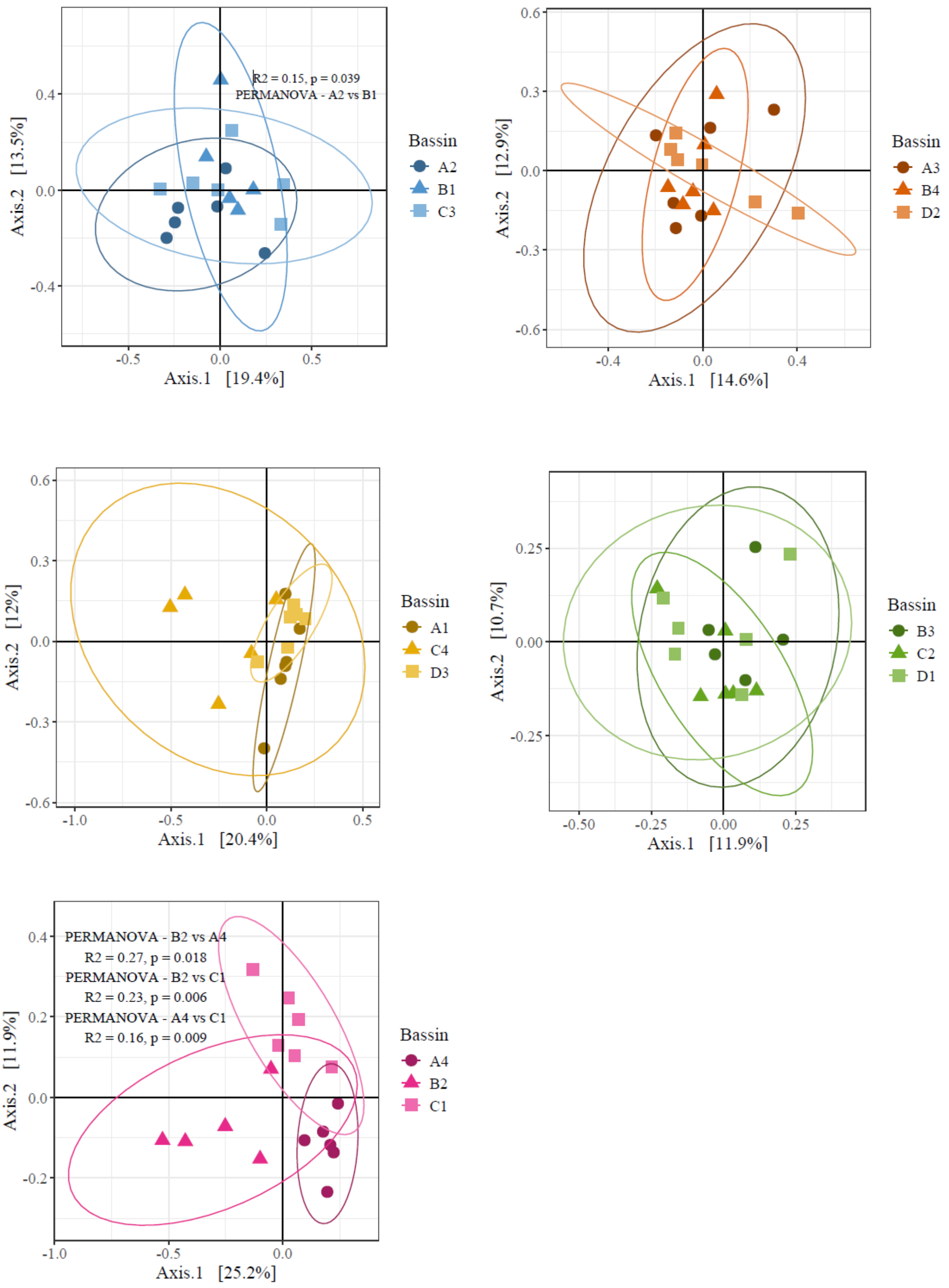

Figure S3: PCoA of the gut microbiome between tanks of the same diet treatment with estimation ellipse of a t-distribution with Weighted UniFrac distances, Blue = Cap, Green = Shr-Cap, Yellow= Cap-Shr, Orange = Shr, Pink = Mix $n_{capelin}$= 17, $n_{capelin\text{-}shrimp}$= 17, n_{mix}= 17, n_{shrimp}= 18, $n_{shrimp\text{-}capelin}$= 17.

Figure S4

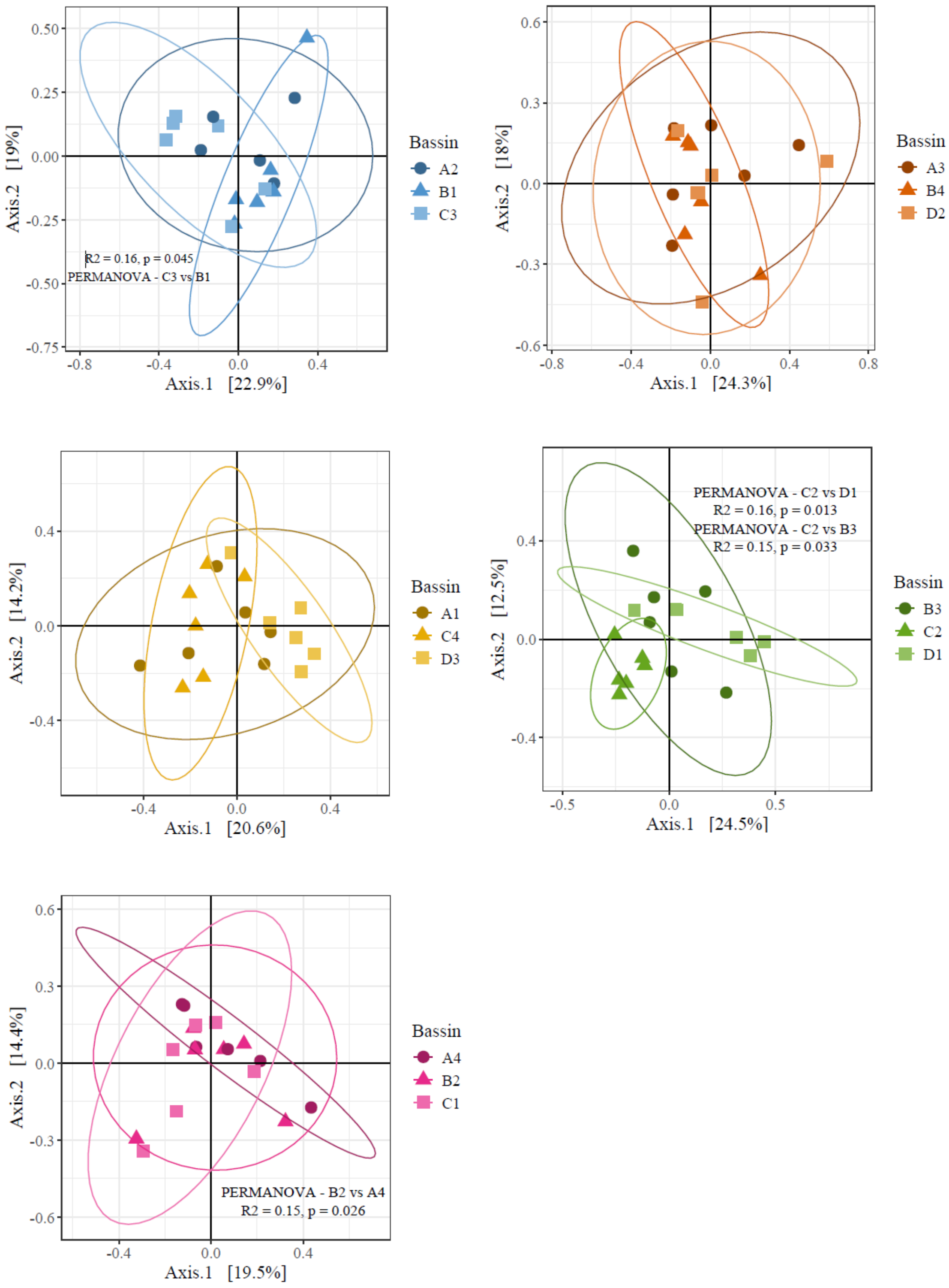

Figure S4: PCoA of the blood microbiome between tanks of the same diet treatment with estimation ellipse of a t-distribution with Weighted UniFrac distances, Blue = Cap, Green = Shr-Cap, Yellow= Cap-Shr, Orange = Shr, Pink = Mix, $n_{capelin}$= 17, $n_{capelin\text{-}shrimp}$= 18, n_{mix}= 18, n_{shrimp}= 18, $n_{shrimp\text{-}capelin}$= 18.

Figure S5

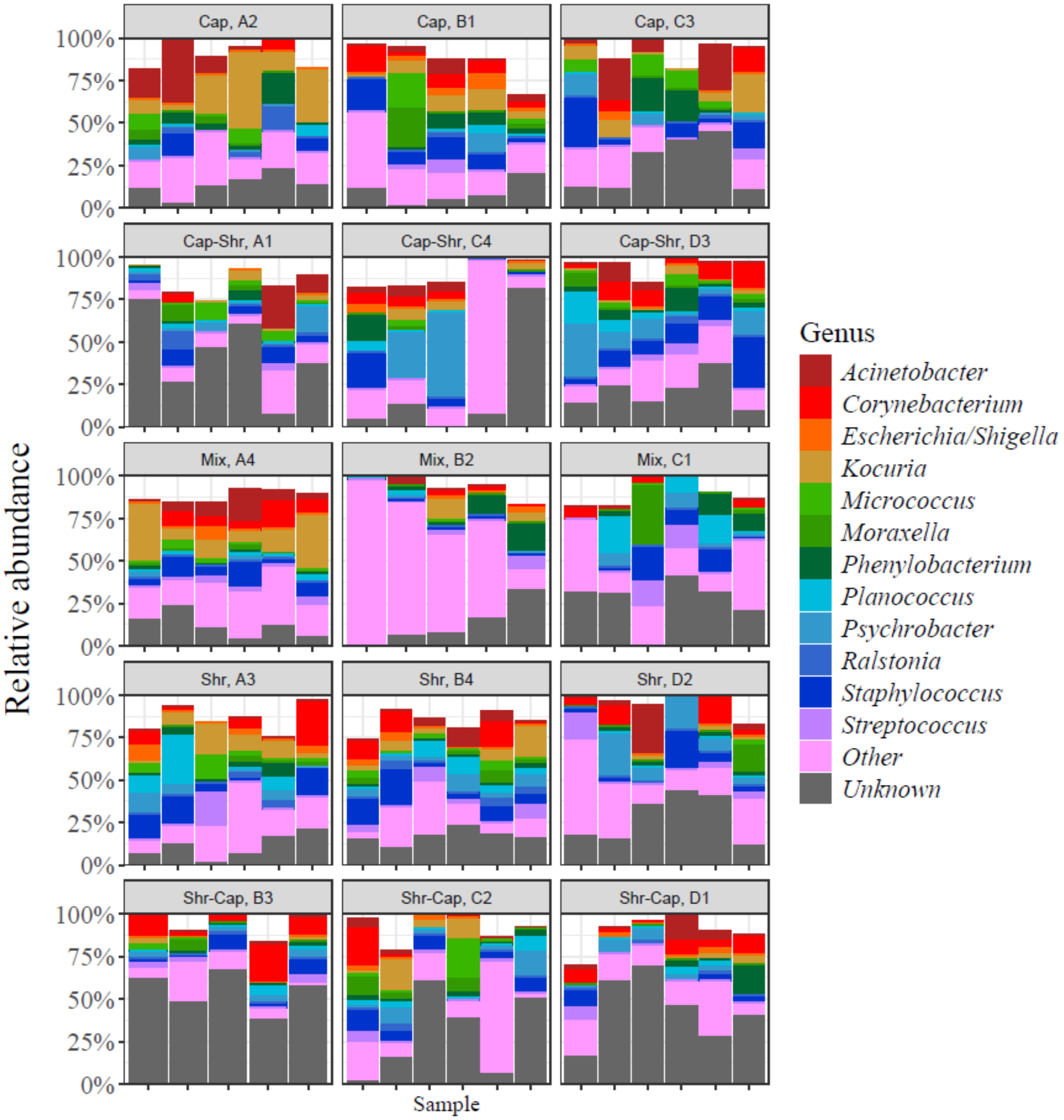

Figure S5: Individual relative abundance of the main shared genera in the Redfish's gut microbiome, n_{gut}=86, prevalence = 20%, detection threshold = 1%.

Figure S6

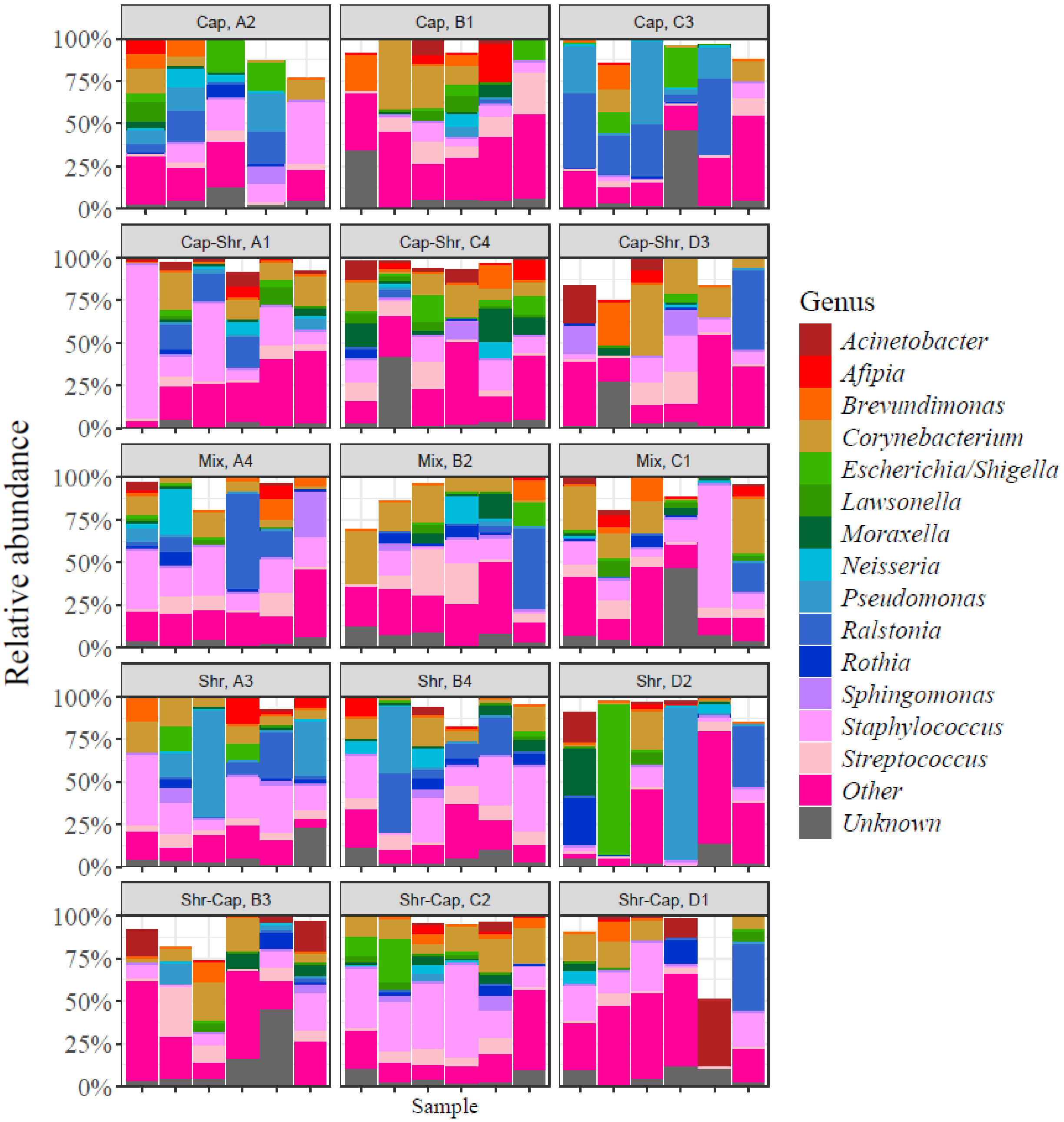

Figure S6: Individual relative abundance of the main shared genera in the Redfish's blood microbiome, n_{blood}=89, prevalence = 20%, detection threshold = 1%.

Figure S7

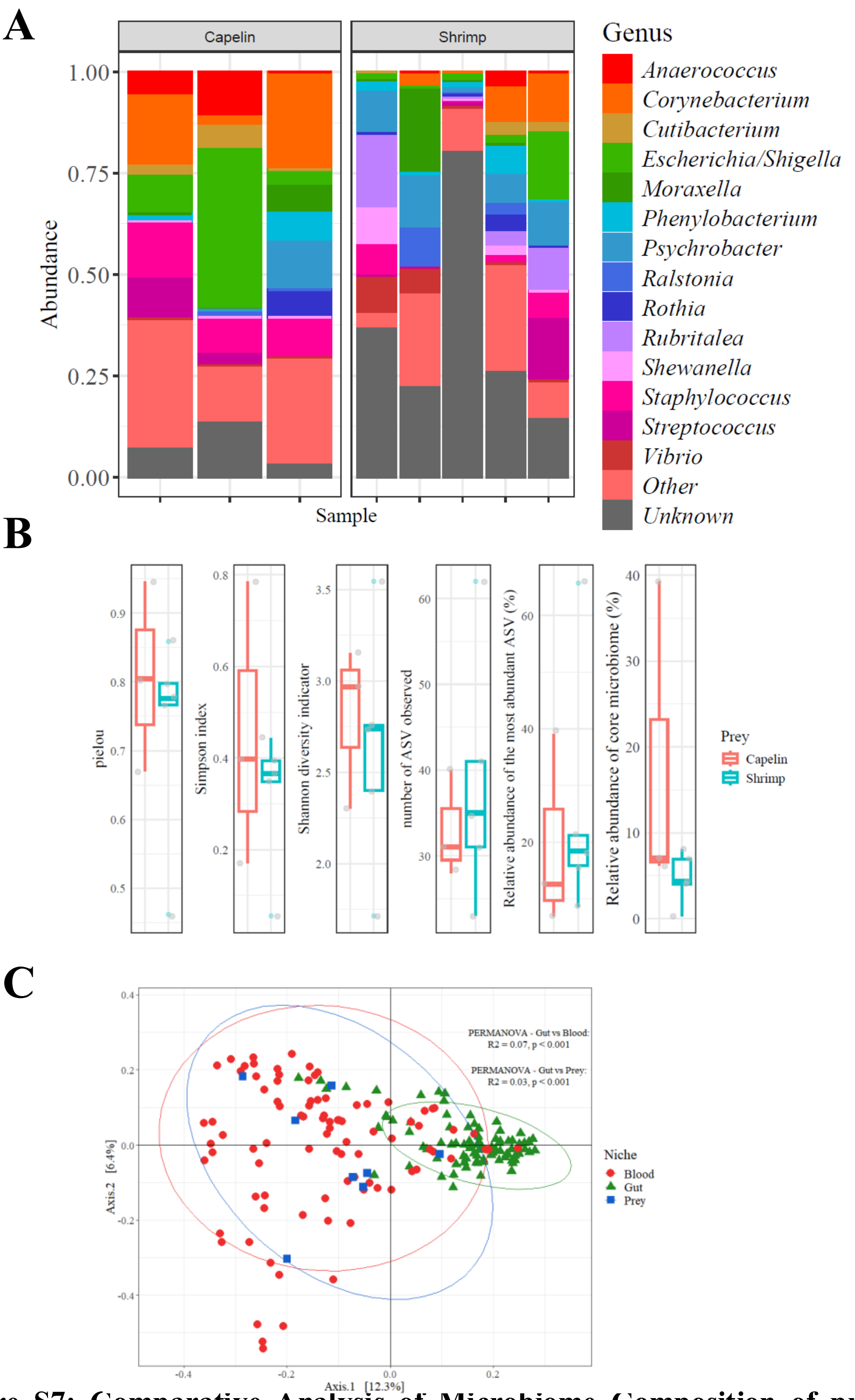

Figure S7: Comparative Analysis of Microbiome Composition of prey. (A) Relative abundance of various bacterial genera in whole extracts of capelin and shrimp. The abundance is normalized such that the total abundance for each sample sums to 1. **(B)** Key diversity metrics and the relative abundance of the most dominant amplicon sequence variants (ASVs) in the prey. **(C)** PCoA between the niche and the prey used in the 6-month experiment, with an estimated ellipse of a t-distribution using Weighted UniFrac distances: red circle = blood, green triangle = gut, blue square = prey; gut, n = 86; blood, n = 89; prey, n = 8. =89.

Figure S8

Ralstonia pickettii strain CN4 16S ribosomal RNA gene, partial sequence

Sequence ID: MW193378.1 Length: 1443 Number of Matches: 1

Range 1: 86 to 773 GenBank Graphics ▼ Next Match ▲ Previous Match

Score	Expect	Identities	Gaps	Strand
1162 bits(629)	0.0	669/688(97%)	3/688(0%)	Plus/Plus

```
Query  18   ATAC-TCGG-ACGTGCCCTG-GGTGGGGGATAACTAGTCCAAAGATTAGCTGATCCCTTT  74
            |||| |||| ||||||||||  ||||||||||||||||| ||||||||||| || ||
Sbjct  86   ATACATCGGAACGTGCCCTGTAGTGGGGGATAACTAGTCGAAAGATTAGCTAATACCGCA  145

Query  75   TACGAGCTGCCGGTGAAAGTGGGGGACCGCACGGCCTCCTGTTATAGGAGCGGCCGATGT  134
            ||||| |||  |||||||||||||||||||| |||||| || ||||||||||||||||||
Sbjct  146  TACGACCTGAGGGTGAAAGTGGGGGACCGCAAGGCCTCATGCTATAGGAGCGGCCGATGT  205

Query  135  CTGATTAGCTAGTTGGTGGGGTAAAGGCCCGCCAAGGCGACGATCAGTAGCTGGTCTGAG  194
            |||||||||||||||||||||||||||||| |||||||||||||||||||||||||||||
Sbjct  206  CTGATTAGCTAGTTGGTGGGGTAAAGGCCCACCAAGGCGACGATCAGTAGCTGGTCTGAG  265

Query  195  AGGACGATCAGCCACACTGGGACTGAGACACGGCCCAGACTCCTACGGGAGGCAGCAGTG  254
            ||||||||||||||||||||||||||||||||||||||||||||||||||||||||||||
Sbjct  266  AGGACGATCAGCCACACTGGGACTGAGACACGGCCCAGACTCCTACGGGAGGCAGCAGTG  325

Query  255  GGAAATTTTGGACAATGGGCGAAAGCCTGATCCAGCAATGCCGCGTGTGTGAAGAAGGCC  314
            || |||||||||||||||||||||||||||||||||||||||||||||||||||||||||
Sbjct  326  GGGAATTTTGGACAATGGGCGAAAGCCTGATCCAGCAATGCCGCGTGTGTGAAGAAGGCC  385

Query  315  TTCGGGTTGTAAAGCACTTTTGTCCGGAAAGAAATGGCTCTGGTTAATACCTGGGGTCGA  374
            ||||||||||||||||||||||||||||||||||||||||||||||||||||||||||||
Sbjct  386  TTCGGGTTGTAAAGCACTTTTGTCCGGAAAGAAATGGCTCTGGTTAATACCTGGGGTCGA  445

Query  375  TGACGGTACCGGAAGAATAAGGACCGGCTAACTACGTGCCAGCAGCCGCGGTAATACGTA  434
            ||||||||||||||||||||||||||||||||||||||||||||||||||||||||||||
Sbjct  446  TGACGGTACCGGAAGAATAAGGACCGGCTAACTACGTGCCAGCAGCCGCGGTAATACGTA  505

Query  435  GGGTCCAAGCGTTAATCGGAATTACTGGGCGTAAAGCGTGCGCAGGCGGTTGTGCAAGAC  494
            ||||||||||||||||||||||||||||||||||||||||||||||||||||||||||||
Sbjct  506  GGGTCCAAGCGTTAATCGGAATTACTGGGCGTAAAGCGTGCGCAGGCGGTTGTGCAAGAC  565

Query  495  CGATGTGAAATCCCCGAGCTTAACTTGGGAATTGCATTGGTGACTGCACGGCTAGAGTGT  554
            ||||||||||||||||||||||||||||||||||||||||||||||||||||||||||||
Sbjct  566  CGATGTGAAATCCCCGAGCTTAACTTGGGAATTGCATTGGTGACTGCACGGCTAGAGTGT  625

Query  555  GTCAGAGGGGGGTAGAATTCCACGTGTAGCAGTGAAATGCGTAGAGATGTGGAGGAATAC  614
            ||||||||||||||||||||||||||||||||||||||||||||||||||||||||||||
Sbjct  626  GTCAGAGGGGGGTAGAATTCCACGTGTAGCAGTGAAATGCGTAGAGATGTGGAGGAATAC  685

Query  615  CGATGGCGAAGGCAGCCCCCTGGGATAACACTGACGCTCATGCACGAAAGCGTGGGGAGC  674
            ||||||||||||||||||||||||||||||||||||||||||||||||||||||||||||
Sbjct  686  CGATGGCGAAGGCAGCCCCCTGGGATAACACTGACGCTCATGCACGAAAGCGTGGGGAGC  745

Query  675  AAACAGGATTAGATACCCCGGTAGTCCA  702
            |||||||||||||||||| |||||||||
Sbjct  746  AAACAGGATTAGATACCCTGGTAGTCCA  773
```

Figure S8: BLAST (Basic Local Alignment Search Tool) results obtained by Sanger sequencing following semi-nested PCR analysis using primers specific for *R. pickettii.* of an amplicon for species verification of *Ralstonia*.

Supplementary Table S1

Table S1: RDA results of the measured factors analyzed with gut and circulating microbiome taken separately

Niche	Factors	Degree of freedom	Variance	F statistics	p-value
**Gut**	Length at the start	1	0.895	0.9341	0.6474
	Length at the end	1	1.148	1.1976	0.1119
	Weight at the start	1	0.859	0.8964	0.7283
	Weight at the end	1	1.120	1.1694	0.1299
	Gonad's weight	1	1.160	1.2103	0.1209
	Liver's weight	1	1.053	1.0993	0.2498
	Length growth per day	1	0.928	0.9742	0.5094
	IGS	1	0.826	0.8625	0.7842
	IHS	1	0.814	0.8498	0.8042
	Fulton's K	1	0.803	0.8380	0.8531
	Sex	2	1.955	1.0199	0.4855
	Diet treatment	4	5.623	1.4670	0.0009 (***)
	Basin	10	11.517	1.2019	0.0009 (***)
	Residuals	58	55.576		
**Blood**	Length at the start	1	0.324	0.9050	0.5904
	Length at the end	1	0.275	1.1976	0.1119
	Weight at the start	1	0.244	0.8031	0.8282
	Weight at the end	1	0.305	1.0043	0.4216
	Gonad's weight	1	0.303	0.9984	0.4386
	Liver's weight	1	0.265	0.8741	0.7013
	IGS	1	0.243	0.8011	0.8291
	IHS	1	0.374	1.2312	0.1389
	Fulton's K	1	0.4101	1.3514	0.0509 (.)
	Sex	2	0.481	0.7932	0.6763
	Diet treatment	4	1.519	1.2515	0.0150 (*)
	Basin	10	3.990	1.3149	0.0009 (***)
	Residuals	61	18.510		
